# RELATIONSHIP BETWEEN QUADRICEPS ECHOGENICITY AND KNEE EXTENSOR STRENGTH IN OLDER ADULTS: A SYSTEMATIC REVIEW

**DOI:** 10.1093/geroni/igad104.3563

**Published:** 2023-12-21

**Authors:** Michael Harris-Love, Dustin Oranchuk, Katie Boncella, Ben Rush, Stephan Bodkin

**Affiliations:** University of Colorado, Centennial, Colorado, United States; University of Colorado-Anschutz Medical Campus, Aurora, Colorado, United States; University of Colorado-Anschutz Medical Campus, Aurora, Colorado, United States; University of Colorado-Anschutz Medical Campus, Aurora, Colorado, United States; University of Utah, Salt Lake City, Utah, United States

## Abstract

The clinical availability of assessing muscle quality through diagnostic ultrasound provides a viable screening tool for geriatric syndromes that impair mobility. However, relationships between muscle echogenicity and clinical assessments require further inquiry. The authors aimed to systematically identify and synthesize the literature regarding the relationship between quadriceps echogenicity and maximal knee extensor strength in older adults. A total of 12 extracted articles (N=701, 73.6±5.4 years) independently assessed the relationship between quadriceps echogenicity and peak knee extensor torque. Correlations were weak to moderately negative between knee extensor strength and rectus femoris echogenicity in older adults (mean ±standard deviation; r=-0.398±0.159 [range=-0.681 to -0.080]). While the limited number studies did not warrant advanced analyses, but it appears that the echogenicity of the vastus medialis (study n=3, r=-0.426±1.97 [range=-0.640 to -0.253]) and vastus lateralis (n=3, r=-0.411±0.095 [range=-0.511 to -0.322]) may be superior to the vastus intermedius (n=4, r=-0.341±0.166 [range=-0.484 to -0.120]) and rectus femoris (n=7, r=0.290±0.140 [range=-0.460 to -0.080]) for predicting knee extension strength in older adults. Furthermore, averaging across multiple quadriceps muscles generally improved correlations (n=4, r=-0.544±0.154 [range=-0.681 to -0.334]). Overall, correlations with knee extensor strength were stronger when echogenicity was corrected for subcutaneous fat thickness (n=3, r=-0.491±0.04 [range=-0.521 to -0.453]). However, results regarding fat correction were inconsistent within studies (n=2, uncorrected r=-0.120 and -0.527 versus corrected r=-0.500 and -0.453, respectively). Future studies should aim to report correlations for all quadriceps muscles and for both corrected and uncorrected echogenicity values to allow for more thorough analytical approaches.

